# Recycling of Cigarette Butts in Fired Clay Bricks: A New Laboratory Investigation

**DOI:** 10.3390/ma13030790

**Published:** 2020-02-09

**Authors:** Halenur Kurmus, Abbas Mohajerani

**Affiliations:** School of Engineering, RMIT University, Melbourne 3000, Australia; abbas.mohajerani@rmit.edu.au

**Keywords:** recycling, cigarette butts, fired clay bricks, energy saving, brick manufacturing

## Abstract

Cigarette butts (CBs) are the most commonly littered waste material in the world. It is estimated that over 5.7 trillion cigarettes are consumed worldwide each year. Consequently, millions of tonnes of highly toxic waste are contaminating the environment. CBs are composed of cellulose acetate filters—a polymer with poor biodegradability—and which, depending upon the environmental conditions, can take many years to decompose. In this study, fired clay bricks were manufactured with 0.5%, 1%, 1.5%, and 2% CBs by mass and tested against control bricks with 0% CBs. The results revealed a decrease in compressive strength from 48.6 MPa for 0% CB content bricks to 30.8 MPa for 1% CB content bricks, and a decrease in dry density with the increase in CB content, from 2114 kg/m^3^ for the control bricks to 1983 kg/m^3^ and 1969 kg/m^3^ for 1% and 2% CB content bricks. The highest value of water absorption appeared for 2% CB content bricks, which reached an absorption rate of 13.1% compared to 9% for the control bricks. The energy required during the firing process was calculated with a saving of up to 10.20%, for bricks incorporating 1% CBs. The thermal conductivity of the samples showed a reduction of 17% from 1.078 to 0.898 W m^−1^·K^−1^ with the addition of 1% CBs. In addition, the manufactured bricks were tested for efflorescence, an initial rate of absorption (IRA), microstructural analysis, and shrinkage. A life-cycle assessment (LCA) is recommended to analyze the environmental impacts of bricks incorporating CBs.

## 1. Introduction

Due to the increasing requirements of environmentally-friendly, lightweight, and low-cost construction materials, many studies have been devoted to finding ways to optimize the performance of the materials used in the building industry, such that the effect on the environment is minimized [1,2]. Ultimately, one-third of the world’s energy consumption is consumed in buildings [3], while 50% of this is lost due to the poor thermal conductivity of the walls [4]. Fired clay bricks are the most conventionally used masonry material around the globe [5]. Their interesting physical, thermal, and mechanical properties allow them to be the ultimate material for the construction of buildings, especially their durability, compactness, and strength [6].

Buildings consume great amounts of thermal energy. During the winter, the internal warmth generated by heating is lost due to the high thermal conductivity of traditional bricks, while, in the summer, the coolness can escape due to the same reasoning. Therefore, it is essential to develop a method to produce thermally-resistant walls, while remaining in the realms of practicality and maintaining the aesthetic appeal of the construction. Hence, enhancing the insulation effectiveness of construction material will significantly lower the impact on the environment. 

Many studies have been conducted to find ways to improve the thermal conductivity of fired clay bricks, with one being the recycling of waste materials composed of high organic content. It was found that the greater the energy consumption during the firing process, the greater the thermal conductivity of the bricks [7,8]. Therefore, the incorporation of additive materials with high organic content may create voids within the structure during the firing process and reduce energy consumption [9]. Wastes have higher heating values (HHV), which are added through self-combustion within the clay mixture, and, hence, less energy is required during the firing process [10]. In doing so, the porosity will increase due to the creation of pores, and the thermal conductivity and density of the fired clay bricks will decrease [11,12]. However, these modifications to the microstructure and composition of the brick will have a negative impact on the mechanical resistance of the material. 

The correlation between density and thermal conductivity is evident [9]. However, it is conceivable to discover situations where there is a variation between thermal conductivity and density [7,13]. The sources of these variations may be the nature and distribution of the pores or the presence of certain mineralogical components [13,14]. A study was completed on 29 samples of clay bricks in which the thermal conductivity was determined. Furthermore, the relationship with their physical, microstructural, and compositional features was evaluated. It was found that mineralogical components, like Ca-rich silicates, quartz, and amorphous phase, play a significant role in decreasing the insulation properties of fired clay bricks, while open pores have a positive effect on thermal conductivity [13].

A number of studies have been conducted on recycling wastes in fired clay bricks to investigate the relationship between the use of additives in fired clay bricks and the micro-pores and nano-pores formed as a result. Such additives include waste sawdust [15], desulfurization slag and basic oxygen furnace aggregates [16], marble powder [17], biomass incineration [18], recycled paper processing residues [4], bio-briquette ash [19], sugarcane bagasse ash [20], and cigarette butts [21,22,23,24,25,26,27]. A recent study revealed the substructures produced by the additive olive pomace (10% weight) that resulted in a 14.4% decrease in thermal conductivity [28]. Comparing this to expanded vermiculite (10% content), a pore-forming material that expands when heated declined from 0.96 to 0.65 W/mK, which is a 32% drop in thermal conductivity [3]. While olive mill solid residue (OMSD) demonstrated the highest decrease in thermal conductivity of 70% with the use of 15% OMSD in fired clay bricks [29].

### Cigarette Butts

**Figure 1:**
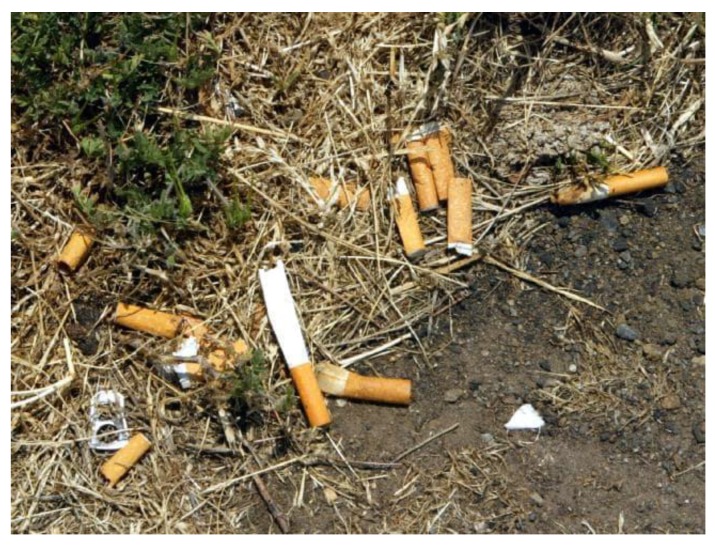
Littered CBs.

Cigarette butts (CBs) are the most commonly littered waste material in the world. The Tobacco Atlas [30] reported that 5.7 trillion cigarettes were consumed worldwide in the year 2016 and that about 97% of the cigarette filters were made up of cellulose acetate filters, which is a modified natural polymer [31]. In Australia, every year, more than 20 billion cigarettes are consumed, and 7 billion are littered into the environment (Figure 1) [32]. Clean Up Australia [33] reported that CBs were the most common source of rubbish collected under the miscellaneous category, which represented 91.5%.

When CBs are littered, harmful chemicals are leached into the environment, which poseleading concerns for the global environment and the quality of urban and aquatic life. In cigarette smoke, it is possible to classify more than 4000 chemical components that are generated during burning or that are distilled from tobacco, of which 69 are carcinogenic [34,35]. Cigarettes generally incorporate cellulose acetate filters, which are designed to fully or partially retain particulate smoke components, including tar and toxic chemicals [36]. Cellulose acetate filters have poor biodegradability and can take up to 18 months to decompose under normal environmental conditions, and, hence, pose a severe dilemma in terms of toxic waste and litter disposal [37,38]. CBs littered on streets are commonly washed away into stormwater drains and end up on beaches, or in rivers and harbors where they leach toxic chemicals [39].

Landfilling and incineration are possible disposal methods for CBs. However, both methods are costly and universally unsustainable. The incineration of CBs can result in the emission of various hazardous substances, and landfilling of CBs will continue to be an environmental hazard regardless of the location of the landfill and can be severely detrimental to the environment and human health [40,41]. Therefore, it is becoming vital to find a practical and effective way to recycle CB waste. An alternative to the growing pollution catastrophe may be the method of recycling CBs into composite building materials such as fired clay bricks.

Mohajerani et al. [27] propose that all CBs can be sustainably recycled in fired clay bricks by incorporating 1% CBs in 2.5% of the world’s brick production. The study involves incorporating various percentages of CBs in clay bricks, including 2.5%, 5%, 7.5%, and 10% [23,24,27]. Experiments have confirmed a steady decrease in dry density and compressive strength with an increase in the CB content of the clay bricks. This decrease in density increases the porosity, which results in an exponential drop in thermal conductivity. Thus, this results in improved thermal performance and greater energy efficiency [26]. The lower energy consumption during the firing process is contributed through the high calorific value of the CBs. CBs have higher heating values (HHV), which are added through self-combustion within the clay mixture, and, hence, less energy is required during the firing process [10]. Moreover, the high temperatures during the firing stage will result in the volatilization of dangerous components and eliminate any toxic compounds [27].

This paper is the continuation of an ongoing study on the incorporation of CBs in fired bricks. In this study, the properties of 0.5%, 1%, 1.5%, and 2% CB bricks by mass (equivalent to approximately 10 kg/m^3^, 20 kg/m^3^, 30 kg/m^3^, and 40 kg/m^3^ CBs) were investigated and compared with controlled fired clay bricks (0 kg/m^3^ CBs). The energy saving percentage during the firing process of bricks and the reduction in thermal conductivity are presented as a function of the CBs present in the mixtures. Additionally, numerous tests were carried out, including water absorption, compressive strength, efflorescence, an initial rate of absorption (IRA), density, shrinkage, and microstructural analysis.

## 2. Materials and Methods

### 2.1. Materials

The soil used in preparing the fired clay bricks was sandy silty clay (MC), which was provided by PHG Bricks Victoria (Victoria, Australia). Geotechnical laboratory tests were conducted on the soil, including the particle size distribution test (PSD), liquid limit, and plastic limit, according to the Australian Standards [42,43,44].

**Figure 2:**
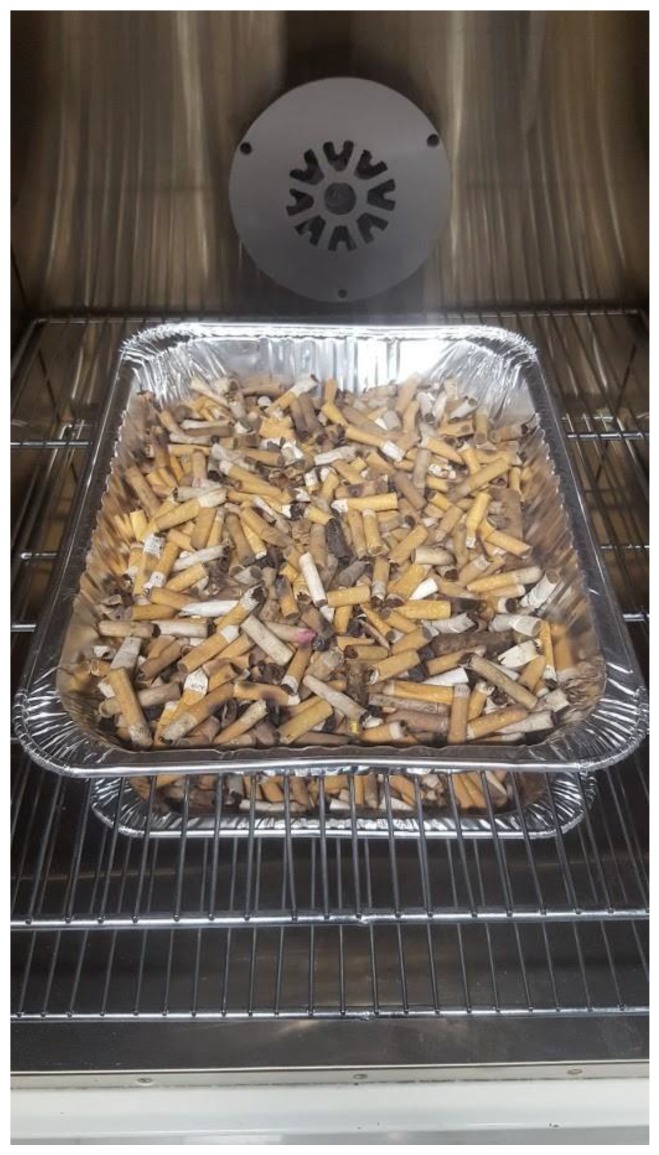
CBs used in this study.

CBs of varying sizes and brands were provided by Buttout Australia Pty Ltd. (Melbourne, Australia), which were collected from dry receptacles. On arrival, the CBs were dried and sanitized at 105° for 24 h in the oven (Figure 2), and then stored in airtight plastic bags.

Chemical and mineral analyses were conducted to determine the major chemical and mineral constituents of the experimental soil using X-ray fluorescence (XRF) (S4 Pioneer, Bruker, Billerica, MA, USA) and an X-ray diffraction analyzer (XRD) (D8 Endeavor, Bruker, Billerica, MA, USA). Thermo-gravimetric analysis (TGA) was carried out using the Perkin-Elmer TGA 8000 instrument (Waltham, MA, USA) to investigate the weight loss of the 0% and 1% CB content clay mixtures. 

The geotechnical properties of the soil used in this study were determined according to the Australian Standards [43,44]. By testing the Atterberg limits, it was found that the liquid limit for the brick soil was 34%, while the plastic limit was 25%, respectively. These values are the average results from three replicate tests.

**Figure 3:**
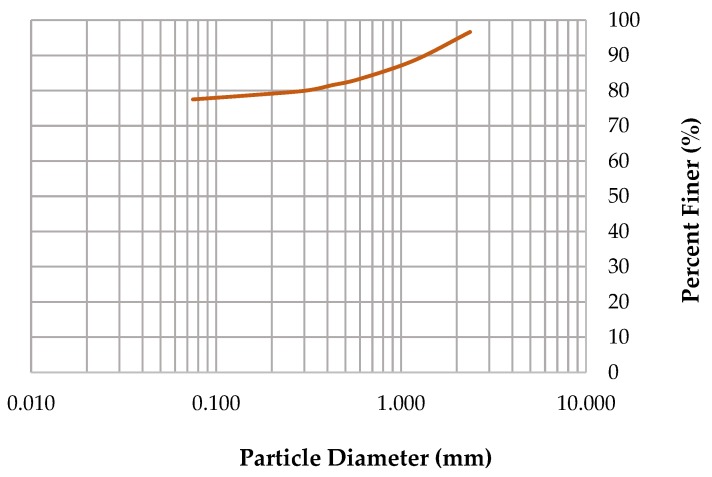
Particle-size distribution curve of brick soil used in this study.

The particle size distribution for the soil sample was determined through sieve analysis and is shown in Figure 3. The brick soil comprised 77.5% fine particles (<75 µm). According to these results, the Unified soil classification of the brick soils is sandy silty clay (MC) [42].

**Figure 4:**
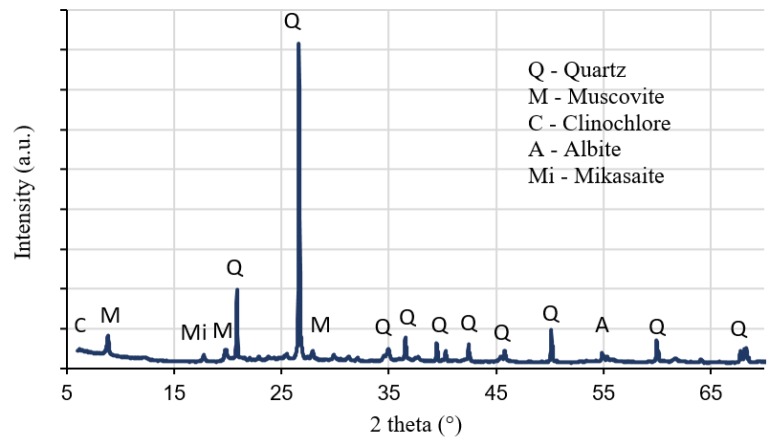
XRD patterns of brick soil.

Chemical analyses were conducted to obtain the chemical composition of the brick soil using XRF. As shown in Table 1, the main compound found in the soil was silicon dioxide, which is followed by aluminium oxide and ferric oxide. XRD was utilized to identify the main crystalline phases on a <75-µm sample of the brick soil (Figure 4). The brick soil displayed Quartz (SiO_2_) as the primary crystalline phase while Muscovite (KAI_2_(AISi_3_O_10_)(F,OH)_2_), Clinochlore (Mg_5_AI(AlSi_3_O_10_)(OH)_8_), Mikasaite (Fe_2_(SO_4_)_3_), and Albite (Na(AlSi_3_O_8_) presented a lower intensity.

**Figure 5:**
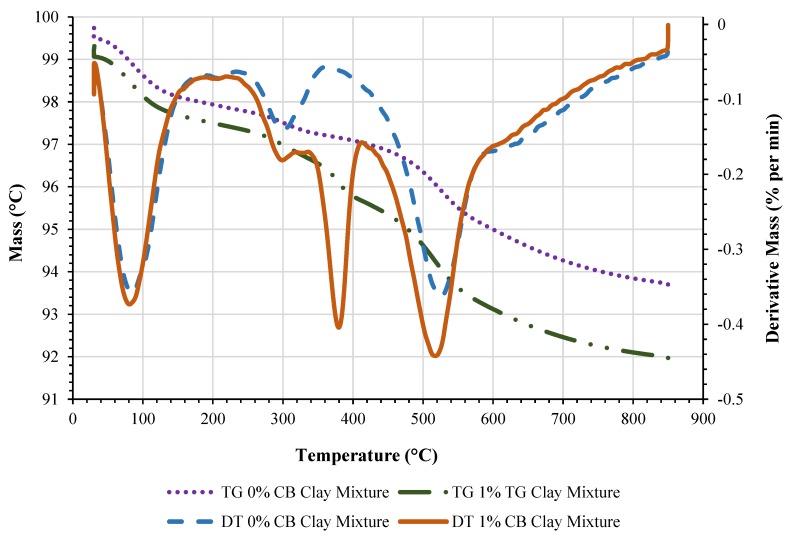
TG and DTA curves of 0% CB content clay mixture and 1% CB content clay mixture.

TGA was performed to monitor and compare the weight loss of the 0% and 1% CB content clay mixtures while the samples were exposed to a constant rate of heat. The samples were heated from 30 °C to 850 °C at 20 °C/min in an air atmosphere with a purge rate of 15 mL/min. Figure 5 presents the thermogravimetric (TG) and differential thermal (DT) curves for 0% and 1% CB content clay mixtures, respectively. The 0% and 1% CB content clay mixtures demonstrated mass loss characteristics of 6.1% (0% CB content clay mixture) and 7.3% (1% CB content clay mixture). The initial decrease occurred at 150 °C. This is due to the evaporation of water within the mixture. While a majority of the mass loss occurred at 150–400 °C and 500–600 °C, during these ranges, organic matter is burned and water molecules of the chemical compounds are released. The final mass loss occurred between 600–800 °C. This can be related to the decomposition of carbonates and sulphides. The 1.2% difference in mass loss between the two samples could be due to the high volatile organic content present in the 1% CB content clay mixture that burnt off during the high temperatures.

### 2.2. Brick Sample Preparation

The bricks were prepared incorporating varying percentages of cigarette butts and a control sample with 0% CBs to investigate the mechanical and physical properties of the bricks. The mixes were categorized by the percentage of CBs by the mass of the brick. CB (0.0) was the control sample and consisted of no CBs. CB (0.5) consisted of 0.5% CBs by mass. CB (1.0) consisted of 1% CBs by mass, CB (1.5) consisted of 1.5% CBs by mass, and CB (2.0) consisted of 2% CBs by mass. 

**Figure 6:**
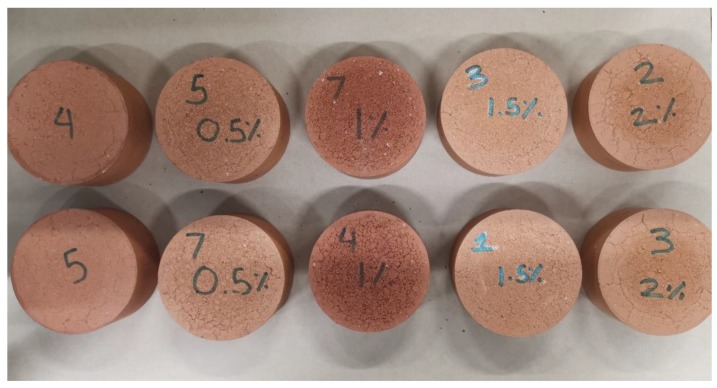
Some of the samples prepared in this study.

The soil and CB mixtures were prepared using a Hobart mechanical mixer for a duration of 25 min with a 15.5% water content. Then it was placed in airtight plastic bags for 48 h to uniformly distribute the moisture throughout the mix. The mixtures were then compacted in molds of a 100-mm diameter to yield bricks with heights of approximately 50 mm with a compaction pressure of 240 kpa. The specimens were air dried for 24 h before being placed in an oven for 24 h at 105 °C. Once removed from the oven, the samples were then fired in an electric furnace at 1050 °C for 3 h. During each stage of the preparation process, the height, diameter, and weight of the samples were measured using an electric scale and digital caliper. The fired samples were tested for water absorption, compressive strength, efflorescence, initial rate of absorption (IRA), density, shrinkage, thermal conductivity, microstructural analysis, and energy savings, according to the Australian Standards [45] (Figure 6). All the results presented are the average of three replicates.

### 2.3. Energy Saving

The calorific value of a material determines the amount of heat (energy) that is produced by the complete combustion of that material. Therefore, utilizing a material with a high calorific value as a partial replacement material in a mixture with a low calorific value will result in a reduction in the overall required energy for bricks, since additional energy is produced by the constituents of the material when combusted, as compared to a standard mixture. 

In the calculation method [27], the firing energy for the fired clay bricks incorporating CBs was calculated, assuming that 2 MJ·kg^−1^ energy was used for firing the bricks. This value was based on a survey conducted in 1993–1994, which shows that the firing energy consumption is in a range of 2 to 3 MJ·kg^−3^ in Association of Southeast Asian Nations (ASEAN) countries [46]. A laboratory experiment was conducted to determine the calorific value of the CBs. Samples of used CBs were tested, and the calorific value for the CBs was found to be 16.53 MJ/kg. 

The calculations were conducted for the CB bricks by considering the weight of CBs incorporated as well as the calorific value of the cellulose acetate. Therefore, the estimated firing energy saved through the addition of CBs into bricks and compared to the control brick was calculated as follows.

(1) $$\mathrm{Energy}\;\mathrm{Used}\;\mathrm{for}\;\mathrm{Control}\;\mathrm{Clay}\;\mathrm{Brick}\;(\mathrm{MJ})\;Q_{1}=q\times m_{1}$$

Energy Used for Clay—B Brick (MJ) Q_2_ = q × m_2_ − CV × m_3_ (2)

(3) $$\mathrm{Energy}\;\mathrm{Saved}\;(\mathrm{MJ})\;Q_{1}-Q_{2}\;=\;(q\;\times \;m_{1})\;-\;(q\;\times \;m_{2}\;-\;\mathrm{CV}\;\times \;m_{3})$$

(4) $$\mathrm{Energy}\;\mathrm{Saved}\;(\%)\;\Delta E\left(\%\right)=\frac{Q_{1}-Q_{2}}{Q_{1}}\times 100\%\;=\frac{\left(q\;\times \;m_{1}\right)\;–\;\left(q\;\times m_{2}\;-\;\mathrm{CV}\;\times m_{3}\right)}{q\;\times m_{1}}\times 100\%$$

where:

q = energy used for brick firing = 2 MJ/kg;

m_1_ = mass of clay in control clay brick (kg);

m_2_ = mass of clay in clay-CB brick (kg);

m_3_ = mass of CBs in clay-CB brick (kg);

CV = calorific value of cellulose acetate = 16.53 MJ/kg.

### 2.4. Thermal Conductivity

Due to the increasing requirements for energy savings, the thermal insulation properties of building materials, such as bricks, have become significant. The heat loss from a building depends on the thermal conductivity of the materials in the roof and wall. While the thermal conductivity of a brick is the quantity of heat transmitted through the brick. Components that influence the effective thermal conductivity of bricks are generally the geometrical design [47], microstructure, and mineralogical composition [48]. 

As part of these studies, Kadir and Mohajerani [22] developed Equation (5) to estimate the thermal conductivity of brick samples. The thermal conductivity of 256 results of various types of concrete, aggregate, and bricks was obtained to develop an equation using regression analysis as a function of density. Whereby, it was found that K (thermal conductivity) can be represented using its relationship with Dry Density.

(5) $$k\;=\;0.0559e^{\left(0.0014\right)\left(\mathrm{Dd}1\right)}$$

Utilizing Equations (6)–(8), Equation (9) was developed to estimate the energy transfer of the fired clay brick samples. According to Fourier’s Law, we find that the Heat Flow Rate (W) can be expressed as:

(6) $$q\;=\;k\;\times \;A\;\times \;\frac{\Delta T}{L}$$

Since the Heat flow rate is measured in Watts, we can also determine that, through reconfiguring, the Current Intensity formula can be found, which is shown below.

(7) $$q\;=\;\frac{Q}{△T}$$

Hence,

(8) $$\frac{Q}{t}\;=\;k\;\times \;A\;\times \;\frac{\Delta T}{L}$$

Re-arranging the above equations, we see a new formula for energy transfer.

(9) $$Q=\frac{k\cdot A\cdot \Delta T\;}{L}\times t$$

where:

q = heat flow rate (W);

ΔT = T_hot_ – T_cold_ = change in temperature (Kelvin);

Q = energy transfer (Wh);

t = time (h);

k = thermal conductivity (W/mK);

L = thickness of the brick;

A = surface area of the material transferring heat.

## 3. Results and Discussion

### 3.1. Properties of the Fired Clay Bricks

**Figure 7:**
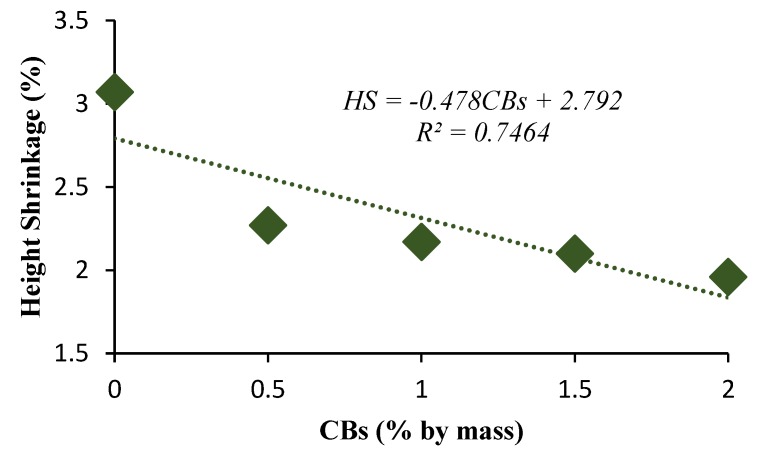
Height shrinkage versus CB percentage by mass.

**Figure 8:**
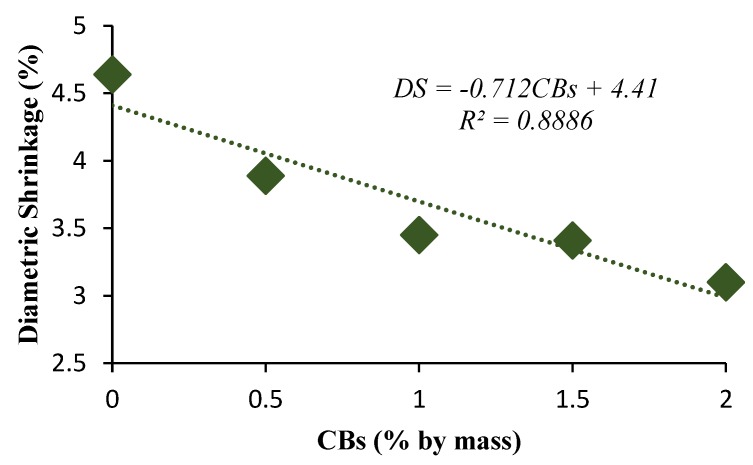
Diametric shrinkage versus CB percentage by mass.

**Figure 9:**
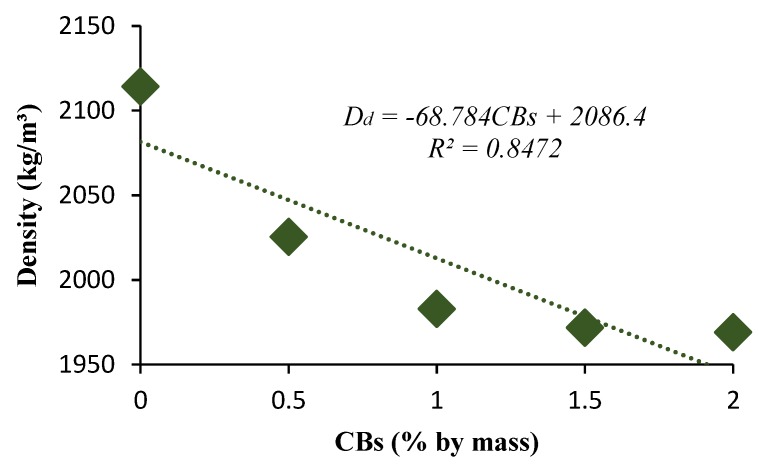
Dry density versus CB percentage by mass.

**Figure 10:**
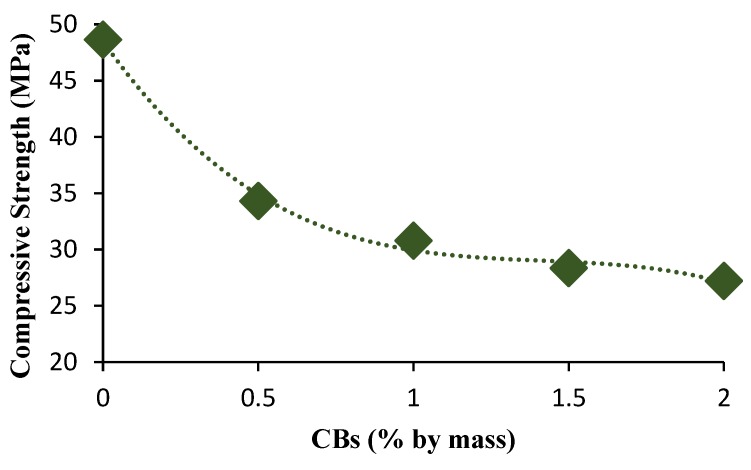
Compressive strength versus CB percentage by mass.

**Figure 11:**
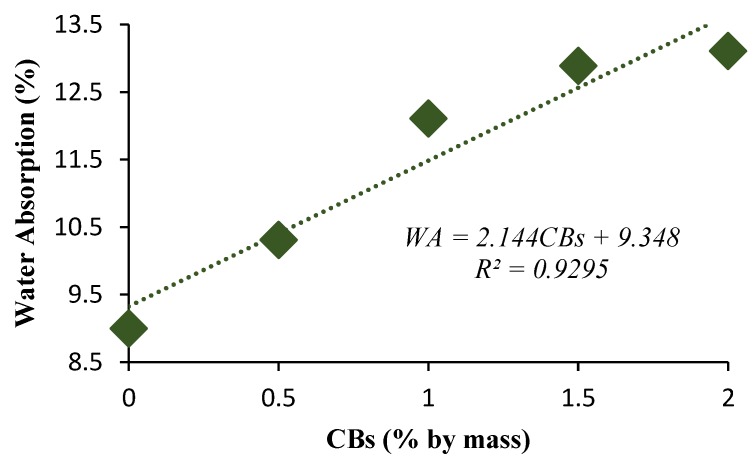
Water absorption versus CB percentage by mass.

**Figure 12:**
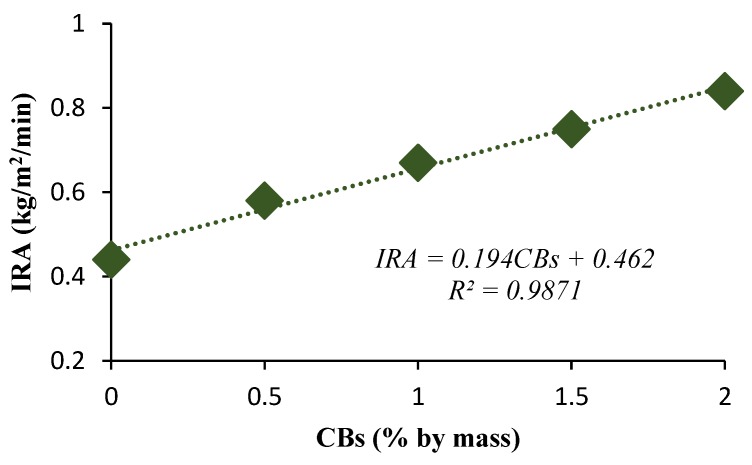
IRA versus CB percentage by mass.

Property tests were conducted for five types of the sample: 0% CBs, 0.5%, 1%, 1.5%, and 2% CBs. The results can be seen in Figure 7, Figure 8, Figure 9, Figure 10, Figure 11 and Figure 12. The dry density of the prepared bricks decreased from 2114 kg/m^3^ for controlled bricks to 1983 kg/m^3^ for bricks with 1% CBs by mass. The addition of CBs resulted in the bricks becoming more porous. The development of pores within the structure of the brick can be associated with the added CBs that burnt off during the firing process as a consequence of the volatile organic content in the CBs.

The compressive strength of the bricks tested decreased from 48.6 MPa for 0% CB content to 30.8 MPa for 1% CB content, and 27.2 MPa for 2% CB content. The minimum recommended compressive strength is 3–5 MPa, and 5–10 MPa for non-load and load-bearing fired clay bricks, respectively [49,50,51]. The reduction in strength was associated with the loss of organic content in the CBs, which were exposed to firing in the furnace. This resulted in the formation of bigger pores due to the CBs that burnt off, which lowered the strength of the CB-amended fired clay bricks. Similar results have been observed with the incorporation of hemp shiv fibers in fly ash-based alkaline mortars. The addition of hemp fibers led to a reduction in compressive strength due to the increase in porosity [52].

**Figure 13:**
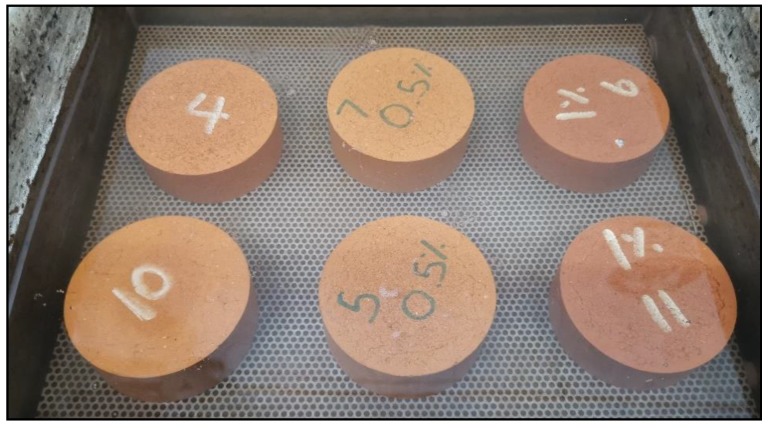
24-hour cold water and 5-hour boiling water test.

The higher percentage of CB content resulted in an increase in water absorption and IRA. The water absorption was measured at 12.1% for 1% CBs by mass compared to 9% for 0% CBs by mass (Figure 13). The greatest value of water absorption was measured at 13.1% for 2% CBs by mass. The IRA was found to be 0.67 kg·m^−2^·min^−1^ for 1% CBs by mass and 0.44 kg m^−2^·min^−1^ for 0% CBs by mass, which falls within the IRA range of 0.2 and 5 kg·m^−2^·min^−1^ [53]. A similar trend was observed with the incorporation of biosolids in fired clay bricks. The increase in the percentage of biosolids resulted in an increase in IRA due to the creation of pores during the firing process [54]. Water absorption and IRA values signify the long-term durability performance of the bricks. Therefore, excessively high values can lead to cracking as a consequence of volume changes.

Drying and firing shrinkage are essential indicators for evaluating the suitability of the added CBs from a durability point of view. The bricks are exposed to high temperatures in the furnace. Therefore, any cracks that occur during drying can result in brick failure during firing. The results revealed a decreasing trend in height and diametric shrinkage with the increase in CB content. The diametric shrinkage values obtained were 4.64%, 3.45%, and 3.1% for 0%, 1%, and 2% CBs by mass bricks, respectively. The decreasing trend may be the consequence of the volatile content present in the CBs that burnt off, which results in a change in size. A similar trend in the variation of shrinkage in fired clay bricks with the increase in waste glass has been reported by Phonphuak et al. [55].

**Figure 14:**
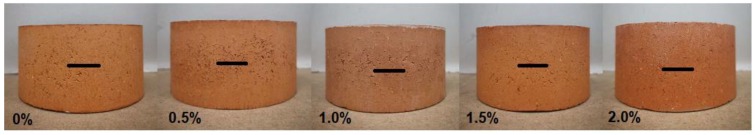
Efflorescence on bricks for mixes with 0%, 0.5%, 1.0%, 1.5%, and 2.0% CBs.

Analysis of efflorescence was carried out for the clay bricks and the clay-CB bricks. Efflorescence is the formation of salt deposits on or near the surface of the brick that causes a change in appearance [56]. For comparison purposes, 10 samples were prepared and sorted into two pairs so that both specimens of each pair had the same appearance. All external faces of each pair of specimens were examined (Figure 14). The results for the potential efflorescence of the control, 0.5%, and 1.5% CB bricks displayed “Nil” observable efflorescence, while there was “Slight” efflorescence for 1%, and “Moderate” efflorescence for the 2% CB bricks, according to the Australian Standards [57].

**Figure 15:**
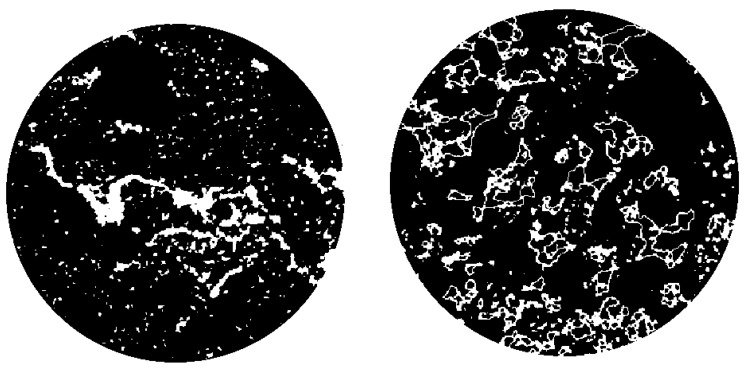
X-ray CT images of 0% (left) and 1% (right) CB content clay bricks.

Figure 15 shows the X-ray computed tomography (CT) images of the 0% and 1% CB content bricks. The white pixels represent the open pores while the black pixels represent the material itself. The increase in CB content resulted in the development of pores of a greater number and size.

### 3.2. Energy Savings

**Figure 16:**
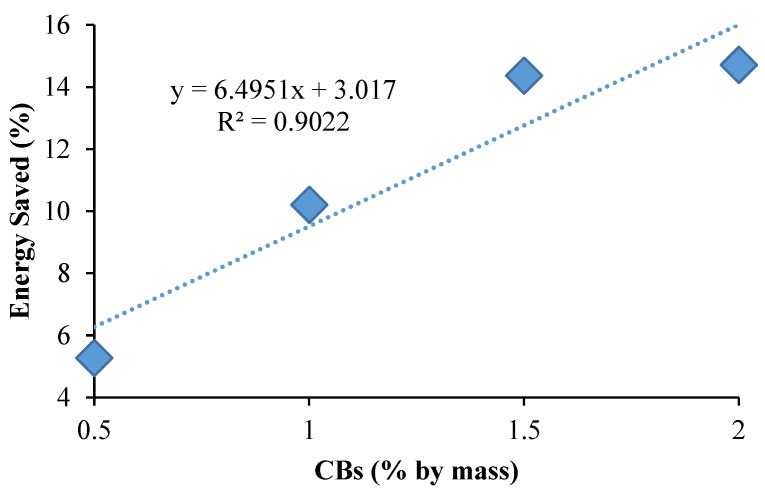
Energy saving versus CB percentage by mass.

Using Equation (4), the estimated amounts of energy saved during the firing process of 0.5%, 1%, 1.5%, and 2% CBs by mass bricks were calculated and are given in Table 2 and Figure 16. The results show that, by firing bricks with 1% CBs by mass, there could be a possible 10.20% energy saving when compared to the control bricks. Furthermore, a 14.71% energy saving can be achieved with an increase in the CB content (2% CB bricks). This is due to the waste having a higher heating value, which is added through self-combustion within the clay mixture during the firing process [9]. 

### 3.3. Thermal Conductivity

**Figure 17:**
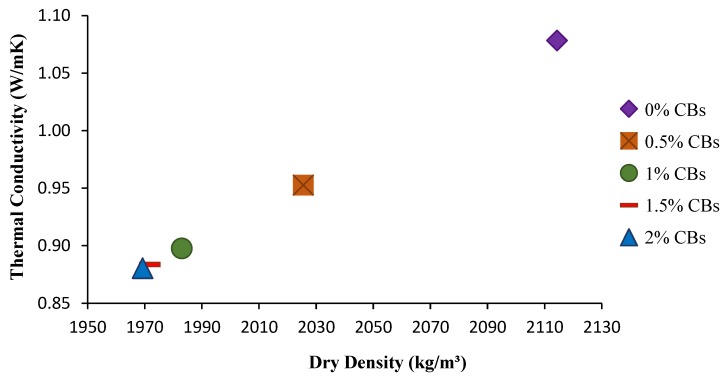
Thermal conductivity of each brick sample.

Using Equation (5), the estimated thermal conductivities for five different brick samples were calculated, and are given in Table 3 and Figure 17. As shown, the increase in CB content results in a reduction in density and thermal conductivity. The incorporation of 1% CBs reduces thermal conductivity by 17%. Lower thermal conductivity is beneficial, as it will decrease the energy consumed for cooling and heating.

**Figure 18:**
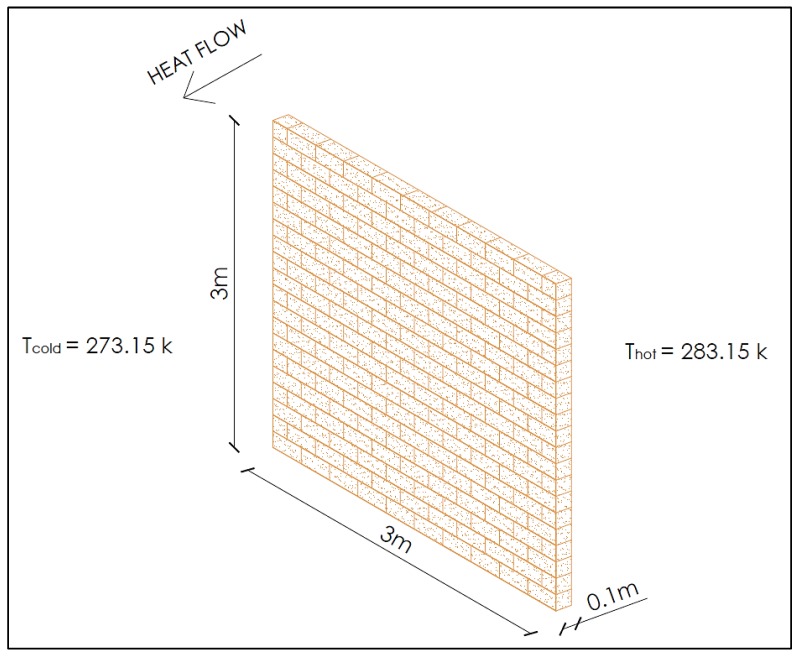
Fired clay brick wall.

Based on the new energy transfer, Equation (9), the energy savings between a fired clay brick wall with 0% CB content and a fired clay brick wall with 0.5%, 1%, 1.5%, and 2% CB content was calculated (Table 4). For the purpose of this example, a 3-m high and 3-m wide wall was considered over a period of 1 year (Figure 18). The thermal conductivity values are taken from Table 3. The temperature of the inner and outer surfaces of the wall is assumed to be 283.15 k and 273.15 k, respectively. Heat flows from the warmer object to the cooler object until they are at the same temperature. The depth of the brick is taken as 0.1 m, and it is assumed that the heat transfer through the wall is steady and that thermal conductivity is constant. 

In Table 4 it can be seen that the increase in CB content corresponds to the reduction in thermal conductivity, and, consequently, a reduction in the energy transfer between the fired clay brick walls. An energy savings of 16.7% was achieved for a brick wall with 1% CB content compared to a 0% CB content wall. This reduction in energy transfer, and, therefore, the energy savings will decrease the costs of heating and cooling. A life-cycle assessment (LCA) is recommended to analyze the environmental impacts of fired clay bricks by incorporating CBs [58].

## 4. Conclusions

This study investigated the properties of 0.5%, 1%, 1.5%, and 2% CB bricks by mass and compared them with those of standard fired clay bricks. Numerous tests were conducted, including water absorption, efflorescence, initial rate of absorption (IRA), density, shrinkage, microstructural analyses, thermal conductivity, and energy savings based on the Australian Standards.

The compressive strength of the bricks tested decreased from 48.6 MPa for 0% CB content to 30.8 MPa for 1% CB mass. The lowest compressive strength value was obtained for the 2% CB content bricks (27.2 MPa). The minimum recommended compressive strength is 3–5 MPa, and 5–10 MPa for non-load and load-bearing fired clay bricks, respectively.

However, the dry density decreased with the increase in CB content from 2114 kg/m^3^ for control bricks to 1983 kg/m^3^ and 1969 kg/m^3^ for 1% and 2% CB content bricks, which produces light-weight bricks. The advantages of light-weight construction material are cheaper to produce and have a lower overall life cycle energy usage and design flexibility. The diametric shrinkage values obtained were 4.64% and 3.45% for the 0% and 1% CBs by mass bricks, respectively, which rises fairly linearly as the CB content increased.

The highest value of water absorption occurred for the 2% CB content bricks, which reached an absorption rate of 13.1% compared to 9% for the control bricks. The initial rate of absorption was found to be 0.67 kg m^−2^ min^−1^ for 1% CBs by mass bricks and 0.44 kg m^−2^ min^−1^ for the control bricks. The increase in CB content resulted in the increase in pores both in size and occurrence, which increased the water absorption rate of the bricks. 

The thermal conductivity was estimated by using a prediction model. The estimations showed that an increase in CB content could result in a significant decrease in the thermal conductivity, and, consequently, result in lower energy costs for cooling and heating. 

The estimated amounts of energy saved during the firing process of 0.5%, 1.5%, 1% CB, and 2% CB content bricks were found using calculations. Approximate savings of 10.20% and 14.71% were seen for 1% and 2% CB content bricks, respectively.

Consequently, bricks incorporating CBs have the potential to provide substantial energy savings and a unique, sustainable recycling solution to a growing global waste catastrophe. However, it is essential to adhere to international regulations and standards regarding the handling and recycling of toxic waste materials. 

The results found in this study complete and confirm the previous findings on recycling CBs in fired clay bricks at RMIT University and the published proposal that all CBs can be sustainably recycled in fired clay bricks by incorporating 1% CBs in 2.5% of the world’s brick production [27].

## Figures and Tables

**Table 1:** Soil chemical properties.

Compound Formula	Brick Soil
SiO_2_	62.84%
Al_2_O_3_	19.95%
Fe_2_O_3_	6.15%
K_2_O	4.87%
Na_2_O	2.46%
MgO	1.62%
TiO_2_	1.19%
CaO	0.27%
Loss on ignition	9.60%

**Table 2:** Calculated value of energy saved during the firing of brick samples.

Mixture Identification %	Mass of Clay Brick, m_1_ Kg	Mass of Clay-CB Brick m_2_ Kg	Mass of CBs in Clay-CB Brick m_3_ Kg	Energy Used for Brick Firing q MJ/kg	Calorific Value CV MJ/kg	Energy Used for Control Brick Q_1_ MJ	Energy Used for Clay-CB Brick Q_2_ MJ	Energy Saved Q_1_ − Q_2_ MJ	Percentage of Energy Saved (Q_1_ − Q_2_)/Q_1_ × 100 %
CB (0.5)	0.758	0.749	0.004	2	16.53	1.52	1.44	0.080	5.27
CB (1.0)	0.758	0.742	0.007	2	16.53	1.52	1.36	0.155	10.20
CB (1.5)	0.758	0.741	0.011	2	16.53	1.52	1.30	0.218	14.36
CB (2.0)	0.758	0.738	0.011	2	16.53	1.52	1.29	0.223	14.71

**Table 3:** Thermal conductivity of all estimated samples.

Mixture Identification %	Dry Density kg/m³	Thermal Conductivity W/mK	Reduction of Thermal Conductivity %
CB (0)	2114	1.078	0
CB (0.5)	2026	0.953	11.66%
CB (1.0)	1983	0.898	16.76%
CB (1.5)	1972	0.884	18.06%
CB (2.0)	1969	0.880	18.35%

**Table 4:** Energy savings between a fired clay brick wall with 0% CB content and 0.5%, 1%, 1.5%, and 2% CB content.

Mixture Identification %	Thermal Conductivity k W/mK	Area of Wall A m²	Change in Temperature ΔT = T_hot_ − T_cold_ kelvin	Time t h	Thickness of Brick L m	Energy Transfer Q kwh	Energy Saved %
CB (0)	1.078	9	10	8760	0.1	8.50	-
CB (0.5)	0.953	9	10	8760	0.1	7.51	11.60%
CB (1.0)	0.898	9	10	8760	0.1	7.08	16.70%
CB (1.5)	0.884	9	10	8760	0.1	6.97	18.00%
CB (2.0)	0.880	9	10	8760	0.1	6.94	18.37%
